# Long-Term Manifestations of COVID-19: A Review

**DOI:** 10.7759/cureus.91492

**Published:** 2025-09-02

**Authors:** Brianna Castellano, Courteney Castellano, Alexandria Sobczak, Deepesh Khanna

**Affiliations:** 1 Medicine, Dr. Kiran C. Patel College of Osteopathic Medicine, Nova Southeastern University, Fort Lauderdale, USA; 2 Foundational Sciences, Dr. Kiran C. Patel College of Osteopathic Medicine, Nova Southeastern University, Fort Lauderdale, USA

**Keywords:** ace-2 receptors, cardiovascular manifestations of covid-19, coronavirus disease, covid-19, covid-19 symptoms, gastrointestinal manifestations of covid-19, long covid syndrome, long-term manifestation of covid-19, neurological manifestations of covid-19, respiratory manifestations of covid-19

## Abstract

Although most coronavirus disease 2019 (COVID-19) cases resolve within a few weeks after the onset of infection, a considerable number of patients still suffer from prolonged or recurrent symptoms evident after weeks or months post-COVID-19 recovery. This paper analyzed the current literature related to long-term manifestations of COVID-19 and aimed to identify the common symptoms reported four weeks or more after the initial onset of the disease. COVID-19 has been shown to have lasting systemic effects on an array of organ systems, such as the lungs, heart, brain, and gastrointestinal systems. Common symptoms include, but are not limited to, fatigue, brain fog, respiratory difficulties, and loss of taste and smell. The impact of COVID-19 on multiple organ systems is thought to be associated with its ability to bind angiotensin-converting enzyme 2 (ACE2) receptors throughout the body and promote cytokine release. This study provides insight into common long-term manifestations of COVID-19. Future studies should look at how long COVID-19 syndrome affects various subpopulations differently.

## Introduction and background

In 2019, the world was taken by storm by the coronavirus disease 2019 (COVID-19) pandemic, and the virus impacted many people across the world. Spread through respiratory droplets, such as sneezing, coughing, or even breathing, COVID-19 is highly infectious and spreads quickly throughout the world [1]. Following exposure, the incubation period of this disease is about 5-14 days, yet individuals are most contagious before developing symptoms. At the beginning of November 2023, 771,679,618 confirmed cases of COVID-19 and 6,977,023 deaths were recorded across the world [2].

During the peak of the COVID-19 pandemic, the most common symptoms at disease onset included cough, fever, myalgia, and altered smell or taste, including anosmia/ageusia [3]. Of the array of symptoms encountered, some of the most notable were asthenia/myalgia (77%) and dyspnea (51%) [4]. Although most patients recover from COVID-19 within a few weeks, many have persistent or recurrent symptoms for months or even over a year following infection. Post-acute COVID-19 is the definition given to symptoms that linger beyond four weeks of primary infection but less than 12 weeks [4]. In an observational study conducted by Seang et al., 30% of patients had complete recovery, 57% had partial recovery, and 13% did not achieve recovery [5]. The Centers for Disease Control and Prevention (CDC) reports that 35% of people in the United States continue to experience COVID-19 symptoms at least two to three weeks following infection [6].

The CDC includes post-acute and post-COVID-19 syndrome in their definition of long COVID, which is persistent acute COVID-19 symptoms beyond four weeks [6]. Subsequently, in October 2021, the World Health Organization (WHO) defined post-COVID-19 conditions as occurring within three months of the onset of COVID-19, with symptoms lasting at least two months [2]. In June of 2022, 18.9% of the population in the United States reported symptoms of long-term COVID-19 after a previous COVID-19 infection [7]. As of April 13, 2024, that number has been reduced to 11.0%. Long COVID syndrome was also seen to be more prevalent among middle-aged Americans, with decreased prevalence noted in ages 18-29 and greater than 60 years [7]. One article reported that 28.9% of the U.S. population with long-term COVID-19 were first infected by severe acute respiratory syndrome coronavirus 2 (SARS-CoV-2) over 12 months prior. Long COVID was more prevalent among adults with comorbidities, female sex, and those who did not receive vaccine boosters [8]. Some factors found to increase the risk of developing long COVID syndrome include increasing age, accompanying chronic diseases, and increased severity of the acute COVID-19 disease [9].

COVID-19 caused by SARS-CoV-2 has been shown to predominantly manifest in the respiratory system, but many individuals report other manifestations. Because of this, long COVID syndrome appears to be a multisystem disease with people having a variety of symptoms affecting multiple organ systems, such as fatigue, brain fog, chest pain, diarrhea, and muscle pain. Some symptoms were dependent on sex and severity of infection, while others were completely independent of such variables [10].

Despite recovery, survivors of COVID-19 infection showed persistent symptoms from underlying organ injury. Some patients have even experienced a worsened/decreased quality of life. Of patients with reported COVID-19 in the United States, 25% stated their day-to-day lives were heavily impacted [8]. There is so much yet to be understood about long COVID syndrome, but it is imperative to continue evaluating and understanding the long-term implications of COVID-19 and understanding similarities and differences among different individuals. Understanding the pathology behind the symptoms of long COVID syndrome may help us with not only predicting the symptoms of long COVID but also preventing and treating the consequences of the disease. With increased knowledge regarding the lasting effects of COVID-19 and the underlying risk factors, improved adherence to preventive behaviors can be achieved. Following the symptomatology of an array of patients months after their COVID-19 infection can lead to a better comprehension of how long COVID manifests, allowing us to learn how to improve and individualize treatment, as well as increase public awareness and prevention.

This review explores the most common manifestations and risk factors associated with long COVID syndrome. We analyzed an array of symptomatology across several demographics and organ systems to provide a comprehensive understanding of how long COVID presents in patients. By exploring several types of literature, mostly primary studies with different populations, we explored common risk factors and predictive factors for long COVID syndrome.

## Review

Methods

A review of post-acute COVID-19 and long COVID syndrome using PubMed, EMBASE, and MEDLINE was performed. Emphasis was placed on articles that sought to identify symptoms of COVID-19 seen in patients at least four weeks after disease onset. Within the research, the main search words used to find articles included Long COVID, Post-COVID-19, and Post-Acute COVID-19. Articles were included if they were peer-reviewed and published in English between January 1, 2020, and December 31, 2023. Other inclusion criteria included patients with new or persistent symptoms at least four weeks after a confirmed or clinically suspected COVID-19 infection. No limitations were made on gender, age, race, or ethnicity. Studies published in a language other than English, coronavirus strains other than COVID-19, unpublished reports, study abstracts, duplicate reporting, review articles, systematic reviews, meta-analyses, and studies published before 2020 were excluded. After an initial search, 31,330 studies were identified. The initial search yielded 11,933 articles, 29 of which were included in the final analysis based on the inclusion and exclusion criteria.

Results

Overview

Of the long-term manifestations of COVID-19, fatigue, respiratory issues, and diarrhea were among the most common. Associated cardiovascular manifestations included arrhythmias, cardiac fibrosis, and increased risk of developing heart failure. Loss of appetite, dyspepsia, loss of taste, and abdominal pain were common gastrointestinal (GI) symptoms. The presenting pulmonary symptoms found in long-term COVID-19 patients have included dyspnea, cough, and chest pain. Fatigue was the most common neurological manifestation, followed by memory and cognitive impairments as well as sleep disorders [3].

GI Manifestations

GI symptoms are common in patients with long COVID syndrome. It is estimated that 15%-29% of patients reported at least one GI symptom six months following the initial infection [11]. Blackett et al. studied two patient cohorts and compared patient specimens of fecal and blood samples acutely and six months after recovering from COVID-19 [11]. Diarrhea was the most common symptom reported (28% of cohort 2). The authors also reported that decreased tryptophan synthesis was correlated with an increase in the severity of the GI symptoms [11].

Diarrhea, constipation, vomiting, and abdominal pain were among the most common GI symptoms of long COVID syndrome [12]. Of the symptoms reported, diarrhea was one of the most common GI manifestations [12]. GI manifestations of long COVID were not found to be related to the initial severity of the COVID-19 infection and could also be present in those even with a mild initial COVID-19 infection [12]. However, the duration of persistent symptoms was longer in participants who had severe COVID-19 as compared to mild or moderate [13].

Carvalho-Schneider et al. [4] followed up adults with non-critical COVID-19 two months after symptom onset. They found that 30 days after onset, 33.1% of patients presented with digestive disorders, and of those, 11.5% still had digestive disorders 60 days post-onset. The GI manifestations at the onset of COVID-19 included mainly diarrhea and vomiting, with the frequency of these symptoms decreasing at 30 and 60 days after the onset of COVID-19. Loss of smell and taste was also reported at the onset of COVID-19 and was found to be the most common symptom at days 30 and 60 post-onset of COVID-19. Symptoms that were still present at day 30 and day 60 were found to be associated with severe COVID-19, age 40-60 years, dyspnea or abnormal auscultation, and hospital admission at symptom onset [4].

A study done by de Miranda et al. [13] monitored outpatient and hospitalized COVID-19 patients with a mean age of 50 years and up to 14 months after the onset of infection. A total of 62.1% of the participants reported pre-existing comorbidities such as hypertension, diabetes, chronic kidney disease, and cancer. Of the GI manifestations, loss of taste or smell (20.1%) and diarrhea (6.8%) were the most common. Almost half of the patients had only one persistent symptom, while 43.8% had two to three symptoms and 8% had four to five symptoms [13].

In addition to common GI manifestations, long COVID syndrome was also associated with unique manifestations. COVID-19 has been identified as a potential “trigger” for the onset of inflammatory bowel disease (IBD). Although uncommon, a small portion of individuals with long COVID developed IBD [14]. Chronic bowel dysfunction and dyspepsia were among other GI symptoms affecting patients months after initial COVID-19 infection [15]. In two case reports of patients who developed IBD at least a month after COVID-19 infection, both experienced diarrhea during acute illness and were women [15,16]. Although the underlying pathogenesis of irritable bowel syndrome (IBS) is multifactorial, IBS after SARS-CoV-2 infection is seemingly related to severe mucosal inflammation in the GI tract as well as altered communication between the gut and the brain [14].

Moreover, COVID-19 syndrome has also been associated with liver injury. Radzina et al. [17] evaluated 90 patients, out of which 34 were in the clinically healthy group, while 56 had SARS-CoV-2 three to nine months before the study. All patients underwent multiparametric ultrasound (mpUS) and were screened for biochemical markers of liver injury. Seventy-six out of the 90 enrolled patients had abdominal magnetic resonance imaging (MRI) and non-contrast-enhanced thoracic computed tomography (CT) scans completed. mpUS identified increased liver stiffness and steatosis, indicating liver injury in the research group. The research group was also observed to have statistically significantly increased liver stiffness with a positive correlation of increased biomarkers of liver injury, such as alanine aminotransferase (ALT) and gamma-glutamyl transferase (GGT). Thirty-four patients in the research group were hospitalized and had a nine times higher chance of having liver steatosis when compared to the control group [17].

Respiratory Manifestations

The presenting pulmonary symptoms found in long-term COVID-19 patients have included fatigue, cough, chest pain, and dyspnea. Zhao et al. [18] analyzed 55 cases with a three-month follow-up period and found that 25% of patients had residual abnormalities of pulmonary function, 71% had radiological abnormalities, and 15% had exertional dyspnea. Common radiograph findings included ground-glass opacities with a mosaic attenuation pattern as well as reticulations and honeycombing [19]. Carvalho-Schneider et al. [4] in their two-month follow-up study reported that dyspnea was one of the most common symptoms at disease onset [4,20]. In a separate cohort of patients previously hospitalized for COVID-19, 81% had dyspnea three months after acute infection, while 24% had abnormal radiographic findings persisting over time. None of these patients had comorbidities such as diabetes, chronic obstructive pulmonary disease (COPD), or asthma, and none of them were smokers [21].

While some pulmonary symptoms seemed to improve over time, others lingered and were still present several weeks after acute COVID-19 infection. Wang et al. [10] in their four-week follow-up study found that although prominent onset symptoms, such as fever, fatigue, and dyspnea, were significantly alleviated, some patients experienced residual symptoms, such as 29.01% reporting cough, 6.11% with chest tightness, and 3.82% with dyspnea [17]. Shah et al. [20] analyzed clinical, radiological, and pulmonary function abnormalities in patients 12 weeks post-COVID-19 onset. After 12 weeks, onset symptoms such as fever, fatigue, and dyspnea were significantly alleviated; however, some residual symptoms, such as cough (29.01%), expectoration (6.11%), chest tightness (6.11%), and dyspnea (3.82%), were still apparent at the time of discharge [21].

Pulmonary function and diffusion capacity of the lungs were lower than expected in the months following COVID-19 infection. In a four-month observational COVID-19 lung study, Guler et al. [19] reported that patients following mild/moderate COVID-19 had normal average pulmonary function, whereas patients following severe COVID-19 had reduced measures of diffusion capacity (DLCO), total lung capacity (TLC), forced vital capacity (FVC), and forced expiratory volume in one second (FEV1) [19]. Lung diffusion capacity (DLCO < 80%) was shown to be reduced significantly in patients, especially those with severe SARS-CoV-2 cases [21,22]. Even at the six-month follow-up, one study showed DLCO to be 76% of predicted. Pulmonary function tests, FEV1 and FVC, were also lower than expected at the six-month follow-up [23].

Neurological Manifestations

Many studies have described the prevalence of neurological manifestations experienced by patients after COVID-19. Of the manifestations noted, fatigue was found to be one of the most common neurological symptoms experienced by patients post-COVID-19 [22-24]. Of 165 patients previously hospitalized for COVID-19, fatigue was the most common symptom reported, followed by memory complaints and sleep disorders [24]. Of 1,230 patients hospitalized with COVID-19 in Wuhan, China, 52% suffered from fatigue and muscle weakness six months after infection [25]. In a study of 189 participants, 42 experienced chronic insomnia three months after COVID-19 infection, and six patients had excessive sleepiness [26]. A study by Dressing et al. [27] identified 31 patients who were admitted for ongoing neurological complaints at least three months after contracting COVID-19. Of these 31 participants, 29% of patients were found to have mild cognitive impairments, while 23% experienced visual memory impairment [27]. Other neurologic symptoms among hospitalized COVID-19 survivors included tremors and motor deficits [26].

Underlying comorbidities and age were both factors that played a role in the presence of neurologic long COVID-19 symptoms [26]. Additional studies further supported that certain symptoms corresponded to the initial severity of COVID-19 infection as well as underlying comorbidities, such as memory and attention deficits, especially in older patients, while other symptoms, such as peripheral neuropathy, were independent of the severity of illness [24]. Increased body temperature during acute COVID infection, as well as decreased SpO_2_, was a predictive factor for psychological and cognitive impairments [28]. The female sex also had a significant effect on physio-affective symptoms as evidenced by the increased prevalence of symptomatic fatigue in women [26,28].

In the months following their COVID-19 infection, many individuals also struggled with mental health concerns such as anxiety and depression. In a cohort study in China, 26% of participants suffered anxiety and depression months after being discharged from the hospital with COVID-19 [25]. After measuring depression and cognition symptoms using the Montgomery-Asberg Depression Rating Scale (MADRS) and Montreal Cognitive Assessment (MoCA), a significant difference was found between patients with long COVID syndrome compared to uninfected healthy controls [29]. Long COVID patients also had increased gray matter volume in the hippocampus, amygdala, basal ganglia, and frontotemporal areas in comparison to healthy participants [29].

COVID-19 can result in a pathology known as post-viral cerebellar ataxia. Although uncommon, there have been various cases reported of cerebellar dysfunction following the recovery of acute COVID-19. For example, a 62-year-old man developed ataxia and scanning speech after an acute COVID-19 infection. All other etiologies of cerebellar ataxia were ruled out, along with any pre-existing cerebellar disease [30]. In addition, cerebral MRI imaging showed mild cerebellar atrophy [30]. After nearly five months and the initiation of steroid treatment, his ataxia finally improved and nearly resolved [30]. Another case report portrayed a 70-year-old male epileptic patient who developed post-infectious cerebellar ataxia five weeks after an acute COVID infection. This patient presented with severe imbalance, tremors, and difficulty walking [31]. Overall, these rare presentations of cerebellar ataxia following COVID-19 suggest a link between the two pathologies, but the mechanism of the relationship needs to be explored further.

Some cases of Guillain-Barré syndrome (GBS), a rare autoimmune inflammatory neuropathy that is usually preceded by an infection, have also been associated with COVID-19. The classic form of GBS, which is known as acute inflammatory demyelinating polyneuropathy (AIDP), has been most commonly associated with COVID-19. This form is characterized by ascending weakness along with loss of sensory function and deep tendon reflexes. Zubair et al. [32] summarized two cases of GBS associated with COVID-19. One case of a 32-year-old man with no pertinent medical history and a confirmed COVID-19 infection was admitted to the ICU and developed acute respiratory distress syndrome (ARDS) that was further complicated by a GI bleed. After the lengthened ICU stay, he had significant generalized weakness and over the next five days developed paresthesia in the lower extremities, resulting in atrophy of his leg muscles along with loss of strength. He ultimately developed severe axonal sensorimotor polyneuropathy, as shown by the electromyography (EMG) and nerve conduction studies (NCSs) conducted. CSF analysis further proved the diagnosis. In addition, a 61-year-old man with diabetes and severe lumbar stenosis with no past medical history of neuropathy presented with generalized weakness and diarrhea that ultimately resulted in a prolonged ICU stay. After 60 days of the initial admission to the hospital, he developed gait instability and new-onset leg weakness with hand involvement that quickly followed. The sensory and motor findings included absent vibration sensation, loss of deep tendon reflexes, and loss of strength in the great toes and patella. Atrophy of the intrinsic hand and foot muscles was also noted. Along with the case of the 32-year-old man, this 61-year-old man was also diagnosed with severe axonal sensorimotor polyneuropathy as shown by his EMG [32].

Cardiovascular Manifestations

It has been hypothesized that COVID-19 affects the autonomic nervous system. Acanfora et al. [33] explored the impact of COVID-19 on vagal activity by studying heart rate variability (HRV). They compared cardiac autonomic activity in 30 long COVID patients and 20 normal COVID patients and concluded that HRV was significantly lower in the long COVID participants compared to the normal COVID participants [33]. HRV is an important marker of dysautonomia, and Acanfora et al. concluded that COVID-19 is linked to causing dysfunction in the autonomic nervous system. A significant association between long COVID and plasma pro-B-type natriuretic peptide (proBNP) concentrations was also found, suggesting elevated myocardial strain or risk of heart failure. In addition to elevated cardiac biomarkers, long COVID patients were also seen to be in a procoagulant state with prolonged elevated D-dimer [33].

Long COVID patients commonly experience symptoms of dysautonomia. A cross-sectional study done at the post-COVID clinic at Assiut University Hospitals, Egypt, analyzed 322 patients [34]. Participants completed the Composite Autonomic Symptom Score 31 (COMPASS‐31) questionnaire, which was used to quantify the severity of the autonomic symptoms. The most common severe autonomic symptoms found in the questionnaire included GI, secretomotor, orthostatic intolerance, and pupillomotor. Autonomic dysfunction was seen as a complication of long COVID in these patients, with 73.6% of participants displaying orthostatic intolerance. These patients had symptoms such as blurred vision, dizziness, and syncope [34].

In a retrospective study done by Dini et al. [35], the most common symptoms among 180 patients exhibiting persistent or new-onset symptoms ≥ 12 weeks after a negative nasal swab test were shortness of breath (52%), followed by heart palpitations (37%) and chest pain (34%). Out of 180 patients, 39 of them met the European Society of Cardiology (ESC) criteria for acute pericarditis. In the patients that had pericarditis, heart palpitations and arrhythmias were more frequent [35].

Persistent endotheliopathy is a common finding among long COVID patients. Compared with healthy controls, COVID-19 patients had a significantly higher thrombin potential and peak thrombin [36]. Fogarty et al. [36] found that plasma FVIII:C levels remained significantly increased compared with controls, and these levels correlated with peak thrombin formation. Thus, these results may highlight the contribution that plasma FVIII levels have on the increased thrombin generation potential in COVID-19 patients. Von Willebrand factor propeptide (VWFpp) levels were also significantly elevated in COVID-19 patients compared to controls. Severe SARS-CoV-2 has been associated with a procoagulant state resulting in life-threatening events such as myocardial infarction and pulmonary embolism [37]. A case of a 31-year-old female patient with no comorbidities who suffered a mild COVID-19 infection developed cardiac arrest and paroxysmal supraventricular tachycardia as a complication of long COVID. There was no history of any other arrhythmias, including supraventricular tachycardia, in the past. CT pulmonary angiography showed evidence of a pulmonary embolism as well as a pulmonary infarct. Elevated D-dimer levels were reported [37]. Usually, pulmonary embolism is seen with cases of severe COVID-19, making this case of a young female patient with a mild COVID-19 infection unusual [37]. In addition, a previously healthy 40-year-old presented to the emergency department with an ECG showing a left bundle branch block three months after recovery from COVID-19 [38]. The patient also had complaints of palpitations and orthostatic intolerance after her recovery, leading up to her ER visit. She also experienced sinus tachycardia, which was seen to be due to postural orthostatic tachycardia syndrome (POTS) [38].

Discussion

COVID-19 emerged in 2019 and shortly led to a global health crisis and pandemic. Millions across the world were impacted, and unfortunately, some have suffered the negative lasting effects of COVID-19 weeks to months post-recovery. Currently, there is still little data on long COVID syndrome and its manifestations. The present review found prolonged symptoms in COVID-19 patients at least four weeks after disease onset. Prevalent symptoms included dyspnea, cough, fatigue, chest pain, and changes in mental status.

In this review, we analyzed the GI, respiratory, cardiovascular, neurologic, and psychiatric manifestations. The entry of SARS-CoV-2 into human cells occurs via angiotensin-converting enzyme 2 (ACE2) receptors, which are found not only in the lungs but also in other organs like the kidneys and liver, providing an explanation for clinical manifestations among various organ systems [11]. Among the GI manifestations of long-term COVID-19, diarrhea and loss of taste were the most common [13]. The mechanisms behind the GI manifestations of long COVID are believed to be due to alterations in the gut microbiome profile, prolonged shedding of virions from the GI tract, or the expression of ACE2 [11]. However, the specific role of these mechanisms is still unclear. One promising theory points to the extensive amount of ACE2 receptors in the intestine [11]. The virus can attach to the ACE2 receptors, enter intestinal cells, and synthesize virions that are released throughout the GI tract [11]. The incidence of post-COVID-19 GI manifestations has also been thought to be due to alterations in the gut microbiome, specifically tryptophan metabolism and 5-hydroxytryptamine (5-HT)-based signaling [11].

The pulmonary manifestations found in long-term COVID-19 have included cough, shortness of breath, chest pain, and dyspnea. The pathophysiology behind lung injury in COVID-19 patients is explained by the fact that type II pneumocytes express the ACE2 receptors that SARS-CoV-2 uses to enter cells [18]. The binding of SARS-CoV-2 to the ACE2 receptors leads to inflammatory responses and cytokine storms resulting in alveolar damage leading to pulmonary scarring and fibrosis [18]. On imaging, residual pulmonary abnormalities and ground-glass opacities depict the scarring and lung fibrosis seen months after COVID-19 infection [18]. The fibrosis and scarring have led to abnormal pulmonary function and impaired diffusion capacity [34].

The most common neurologic manifestations after COVID-19 include fatigue and cognitive impairment. Fatigue was found to be a symptom suffered by patients in both the acute phase and months after viral onset. Some predictive factors of long COVID patients experiencing neurological symptoms include age, the presence of comorbidities, and the severity of the COVID-19 disease [24,26]. Pilotto et al. found that patients with moderate/severe COVID-19 reported a higher number of cognitive symptoms at follow-up and had an increased risk of memory complaints, confusion, and loss of dependency [24]. This association between the severity of COVID-19 and long-term symptoms was only found for neurological manifestations and not for other symptoms such as myalgias and numbness, highlighting the possibility of the central nervous system having a higher vulnerability to SARS-CoV-2 [24]. This could be explained by the fact that severe COVID-19 results in a more severe immune response and, thus, more brain damage [39].

The cardiac manifestations of COVID-19 vary over time and can be characterized as short-term or long-term. Short-term manifestations (<three months) include ECG abnormalities and persistent symptoms like chest pain and dyspnea, suggesting an inflammatory process of the myocardium [35]. Arrhythmias occurring in post-COVID-19 patients have been thought to be associated with right ventricular dysfunction as well as autonomic nervous system dysfunction [35]. Long COVID syndrome has been associated with cardiac injuries such as hypercoagulability, microthrombi, diastolic dysfunction, elevated brain natriuretic peptide (BNP) and D-dimer, and increased myocardial strain [33]. COVID-19 attacks ACE2 receptors, which are commonly expressed in regions responsible for cardiac function [33]. Through adrenal stimulation, COVID-19 can also stimulate the release of vasoconstricting catecholamines such as norepinephrine, altering vascular resistance [33]. The mechanism behind the ongoing cardiac complications following SARS-COV-2 infection is multifactorial, caused by autonomic nervous system dysfunction, vascular injury due to inflammation, and underlying respiratory disease.

A limitation of this study is the inability to define universal post-acute COVID-19 and long-term COVID-19 syndrome manifestations across different clinical studies and to describe the severity of symptoms and their progression. Also, only studies written in English were included in this review, which may show bias against countries in which English is not the primary language. Another limitation of our research is the variance between populations, timelines, and outcomes between studies. A small sample size may also interfere with the results of studies used in this review.

## Conclusions

Following the resolution of acute infection of COVID-19, the virus is persistent, causing symptoms in the months following the onset of infection. Long COVID syndrome causes multisystem impairments such as chest pain, olfactory and gustatory dysfunction, fatigue, and even sleep disturbances. This review describes the various clinical manifestations of long COVID syndrome to highlight the importance of understanding the systemic impact of the virus. Many people worldwide are still suffering the manifestations of COVID-19 months beyond the onset of infection, making long COVID syndrome a phenomenon many researchers are still trying to understand. The reasons for the occurrence of long-term COVID complications in certain populations are still unknown. While the pathology associated with long COVID syndrome is still not entirely understood, inflammation seems to play a pivotal role in the presence and severity of symptoms. One promising explanation involves the activation of ACE2 receptors in an array of organ systems leading to cytokine release and inflammation that can destroy cell tissue. Further understanding the underlying pathological mechanisms of how COVID-19 affects each system is crucial in guiding future treatment in long COVID patients and identifying those at high risk for developing post-COVID complications. Future research needs to explore the pathophysiology of neurological manifestations of the disease, as well as the extent to which long COVID-19 symptoms persist. Long COVID-19 syndrome is a relatively new and poorly understood topic. Therefore, more research is necessary to understand the underlying mechanism and risk factors associated. In the future, it could be beneficial to continue to track patients and see how their symptoms progress. Continued exploration into the risk factors among different patient populations could help further awareness and prevention of COVID-19.

Future studies should focus on the subpopulation variance among long-term COVID-19 symptoms. More research on this topic would help promote a clear definition of post-acute COVID-19 and long-term COVID-19. Because of the nuance of the COVID-19 pandemic, more is being understood daily regarding the lasting impact and manifestations of long COVID-19 syndrome. COVID-19 is a virus with lasting systemic effects that can have a lasting impact on the health and well-being of an array of individuals.
